# Macrophage-induced reactive oxygen species in the initiation of pancreatic cancer: a mini-review

**DOI:** 10.3389/fimmu.2024.1278807

**Published:** 2024-03-21

**Authors:** Heike R. Döppler, Peter Storz

**Affiliations:** Department of Cancer Biology, Mayo Clinic, Jacksonville, FL, United States

**Keywords:** reactive oxygen species, pancreatic cancer, macrophage, initiation, inflammatory

## Abstract

Pancreatic inflammation is a risk factor for the development of pancreatic cancer. Increased presence of inflammatory macrophages can be found in response to a KRAS mutation in acinar cells or in response to experimentally-induced pancreatitis. Inflammatory macrophages induce pancreatic acinar cells to undergo dedifferentiation to a duct-like progenitor stage, a process called acinar-to-ductal metaplasia (ADM). Occurrence of ADM lesions are believed to be the initiating event in tumorigenesis. Here we will discuss how macrophage-induced oxidative stress contributes to ADM and how ADM cells shape the fibrotic stroma needed for further progression.

## Introduction

Chronic pancreatic inflammation (chronic pancreatitis) is a risk factor for pancreatic cancer (1, 2). Macrophages that rapidly increase in numbers after inflammation or in response to an oncogenic KRAS mutation may either originate from tissue resident populations (3, 4) or from external sources such as the peritoneum or blood monocytes (5). Presence of these inflammatory cells can initiate various processes that contribute to lesion formation and progression. For example, in response to macrophage infiltration pancreatic acinar cells can undergo acinar-to-ductal metaplasia (ADM), the dedifferentiation to a progenitor stage with duct-like features (6). ADM lesions are believed to be the initiating lesions for pancreatic intraepithelial neoplasia 1 (PanIN1) (7–10). PanIN1 are precancerous low-grade lesions that form in presence of a KRAS mutation, which occurs in 90-95% of all cases of pancreatic ductal adenocarcinoma (11). In absence of a KRAS mutation, ADM is a reversible process and may contribute to pancreas regeneration after the inflammation resolves (12–14). ADM is triggered by oxidative stress that is generated in acinar cells by macrophage-secreted factors (10, 15–17). ADM cells once formed then crosstalk with different macrophage populations to further drive generation of fibrotic stroma in the lesion microenvironment (18, 19). In the following we will highlight the role of ROS in driving ADM and in progression of ADM lesions with KRAS mutations to precancerous PanIN lesions, but also the contribution of this to the formation of the fibrotic stroma, thus setting the foundation for tumor development.

## Mutant KRAS as an inducer of macrophage attraction

**Figure 1:**
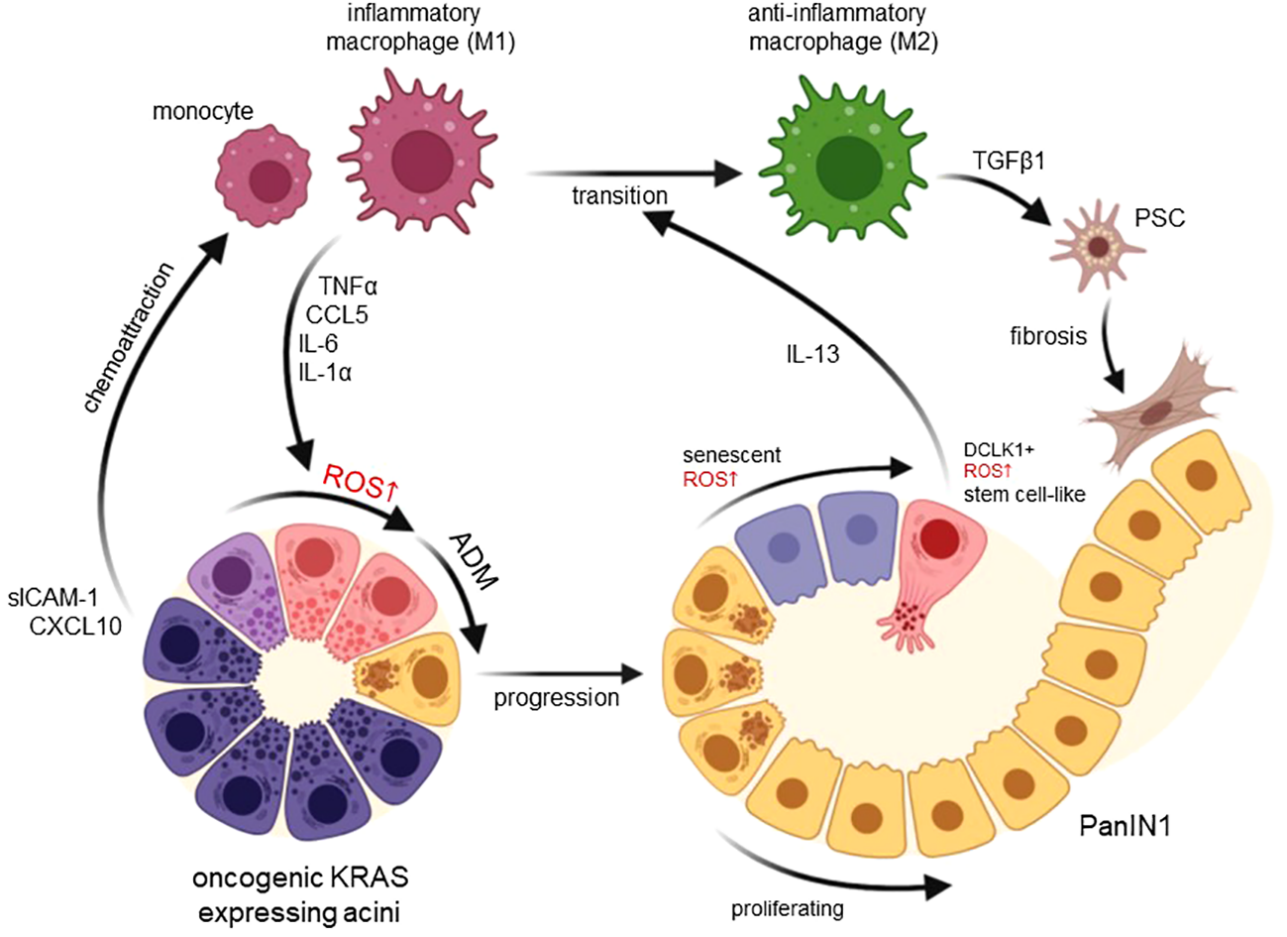
Functions of macrophage-induced ROS in developing pancreatic cancer. Expression of an oncogenic KRAS in pancreatic acinar cells upregulates chemoattractants for monocytes and inflammatory (M1) macrophages including CXCL10 and sICAM-1. M1-secreted factors such as TNFα, CCL5, IL-6 and IL-1α then increase ROS in acinar cells to drive their transdifferentiation to a duct-like phenotype. This process termed acinar-to-ductal metaplasia (ADM) in mice has been shown to be an inducing event for PDA formation when ADM lesions further progress to PanIN lesions. Increasing ROS in PanIN lesion cells can lead to senescence or increased occurrence of DCLK1+ cancer stem cells that produce IL-13. lL-13 mediates a phenotype switch from inflammatory to anti-inflammatory (M2) macrophages in the lesion microenvironment. M2 once abundant can activate pancreatic stellate cells (PSC) to increase fibrosis but also mediate proliferation of lesion cells. Created with BioRender.com.

Pro-inflammatory macrophages are the major immune cell population driving the formation of ADM lesions (10, 16, 17), and their crucial role in this process was demonstrated *in vivo* (in mice) by genetic ablation and by chemical depletion (10, 20). Using the KC (p48^Cre^;LSL-*Kras* ^G12D^) mouse model it was shown that during the development of pancreatic cancer these macrophages accumulate rapidly in ADM regions (21). In KC mice acinar cells with an oncogenic KRAS mutation can upregulate the expression of factors that function as chemoattractants for macrophages or monocytes (21–23). For example, KRAS induces expression of the cell surface glycoprotein Intercellular Adhesion Molecule-1 (ICAM-1, CD54) in acinar cells, and ICAM-1 in its soluble form (sICAM-1) acts as a chemoattractant for inflammatory macrophages (21) (**Figure 1**). Since this leads to focal inflammation, it seems plausible that the initiating macrophage population is recruited locally from a tissue resident population (4).

However, the rapid increase in population density suggests that additional macrophages may be recruited from external sources (5). These could include macrophages from the peritoneum or circulating blood monocytes. Factors to attract both have been demonstrated to be produced in the ADM lesion microenvironment. For example, C-X-C motif chemokine ligand 10 (CXCL10) is produced by ADM lesions in mice and mediates the chemoattraction of inflammatory macrophages to the pancreas, but also enhances their proliferation and maintains their inflammatory identity (22). Other chemoattractants for macrophage are macrophage inflammatory protein-1 (MIP-1) and macrophage inflammatory protein-2 (MIP-2), produced by isolated PanIN lesion cells (22). Further, activated pancreatic stellate cells produce CXCL12 (24), which can act as a chemoattractant for a variety of immune cells, including macrophages. Moreover, the presence of C-C motif chemokine ligand 2 (CCL2), produced by a multitude of cells in the precancerous environment, attracts bone marrow-derived monocytes (23). Once attracted to the ADM/PanIN lesion regions, several cells in their microenvironment produce macrophage proliferation factors. These include M-CSF, produced by activated pancreatic stellate cells (24) and by lesion cells (22).

## Macrophage secreted factors and oxidative stress as drivers of ADM

Pancreatic macrophages can induce acinar-to-ductal metaplasia (10, 17), and ADM lesions that originate from wildtype acinar cells are believed to revert to acini to regenerate the pancreas after the inflammatory stimulus resolves (12, 13, 20). However, in presence of an oncogenic KRAS mutation, ADM lesions progress to low-grade lesions (pancreatic intraepithelial neoplasia, PanIN), which are precursor lesions for PDA (6, 7, 25). The two extreme polarization phenotypes that have been described *in vitro* are pro-inflammatory M1-polarized, classically-activated macrophages (M1, IM) and anti-inflammatory M2-polarized, alternatively-activated macrophages (M2, AAM). *In vivo*, in the inflamed pancreas there is more of a continuous spectrum, however, the majority of macrophages show markers of these two groups, and for simplification we will adhere to these terms (M1 and M2).

Several factors secreted by M1 macrophages have been shown to induce ADM in explant culture. These include TNFα, CCL5 (RANTES), IL-6, and IL-1α (10, 15, 17) (**Figure 1**). Moreover, M2 macrophages can drive ADM through CCL2 (17). All these factors have in common that they induce hydrogen peroxide in acinar cells, which was shown to be the major driver of ADM (16, 17). Hydrogen peroxide most likely originates at the mitochondria, since mitochondrially-targeted catalase or the antioxidant MitoQ can block the initiation of ADM (16).

While several signaling pathways were implicated in driving ADM (reviewed in (6)), a critical event seems to be the upregulation of expression of epidermal growth factor receptor (EGFR) and its ligands TGFα and EGF via ROS-initiated NF-κB signaling in acinar cells (26). Such auto- or paracrine EGFR signaling then is the key event driving both the ADM process and ADM lesion growth (27, 28). Here it also should be noted that macrophages produce other ligands for EGFR including heparin-binding epidermal growth factor-like growth factor (HB-EGF) and amphiregulin (AR), which both may potentiate these effects (29–32).

Although it is still unclear how above macrophage-secreted factors induce ROS at the mitochondria, studies with mitochondrially-targeted antioxidants link ROS responsive signaling cascades and transcriptional activation to ADM. One of the main inducers of ADM downstream of mitochondrial ROS is the PKD1/NF-κB signaling cascade (16, 26, 33). Other transcription factors that have been demonstrated to be predominant drivers of ADM are Notch, Signal Transducer and Activator of Transcription 3 (STAT3), Kruppel-like factor 4 (KLF4) and Nuclear Factor of Activated T Cells 1/4 (NFAT1/4) (34–38). Some of these factors can be activated by macrophage-secreted inducers of oxidative stress. For example, STAT3 activity is upregulated by IL-6 (39) and regulates ADM (35). Others, such as Notch are activated downstream of oncogenic KRAS and the ROS-responsive kinase PKD1 (26, 34) and cooperates with NF-κB to induce ADM (40). However, it should be noted that for most of them a role of direct activation by ROS to drive the ADM processes was formally demonstrated.

## Effects of ROS in low grade lesion cells and role in further lesion progression

Established pancreatic low grade lesion cells (PanIN1) show relatively high levels of oxidative stress as indirectly measured by an increase in 4-hydroxynonenal (4-HNE), a α,β-unsaturated hydroxyalkenal that is produced by lipid peroxidation (41). This increase in oxidative stress correlates well with markers for cellular senescence (42). ROS as a driver of oncogene-induced senescence is established and was implicated in pancreatic lesion cells (42, 43). However, it was also shown that progressing lesion cells increasingly express nuclear factor erythroid 2-related factor 2 (NRF2) (26, 44, 45). NRF2 is a stress-responsive transcription factor that regulates a multitude of genes mediating the antioxidant response and metabolic changes (44, 46, 47). Upregulation of this factor is an important mechanism to overcome ROS-induced damage and senescence in lesion cells and to mediate further progression (**Figure 1**).

A small percentage of lesion cells, however, show exuberant high levels of ROS (48). These cells are positive for DCLK1 and show defective EGFR signaling due to ROS-mediated blockage of vesicle transport (48). DCLK1+ cells express stemness markers such as CD133 and OCT4 (48). Therefore, they have been discussed as stem cells for pancreatic cancer (49) or progenitor cells that promote tumorigenesis (50). Indeed, DCLK1+ cells when isolated and reintroduced in mice, form pancreatic tumors at a faster rate than other lesion cells (49). This may be supported by their secretion of factors that alter macrophage polarity and contribute to generation of the fibrotic microenvironment.

## Alternatively activated macrophages and roles in lesion progression

In presence of an oncogenic KRAS mutation, cells that underwent ADM further progress to PanIN lesion. ADM and PanIN lesion cells can produce IL-13, which induces a polarization switch in inflammatory M1 macrophages to an anti-inflammatory M2 phenotype (19). Major producers of IL-13 are DCLK1+ lesion cells (19). Resulting M2 macrophages, best characterized in mice by expressing arginase (*Arg1*), YM1 (*Chil3*), *Fizz1*, *CD206* and *Trem2* as markers (18, 24), have multiple functions in the progression of lesions. They secrete factors such as TGFβ1 to activate pancreatic stellate cells (PSC) and establish the lesion microenvironment (24), and their ablation either chemically or via neutralization of IL-13 in KC mice leads to a drastic decrease in fibrosis (18, 19). In addition, they secret factors such as TIMP1, IL-4, IL1-ra and CCL2 that act on lesion cells to stimulate their growth via activation of ERK1/2 signaling (17–19, 24). By secretion of CCL2, which generates ROS in acinar cells (17), M2 macrophages also induce ADM in neighboring acinar cells and thus increase abnormal areas in the pancreata of mice. During further progression to PDA, M2 macrophages regulate additional hallmarks of immune escape such as the exclusion of cytotoxic T lymphocytes and fibrosis (51). They also crosstalk with dendritic cells and myeloid derived suppressor cells (MDSC), which inhibit T cell proliferation and induce of T cell death, to further enhance the immune suppressive environment (52, 53). Therefore, targeting this immunosuppressive macrophage population, or initiating their repolarization to an inflammatory phenotype, both are valid strategies to explore for prevention of PDA or to sensitize pancreatic tumors to T cell immunotherapy (19, 51, 54).

## Conclusions

The crosstalk of pancreatic acinar cells with cells of the innate immune system are initiating events in the development of pancreatic cancer (summarized in **Figure 1**). Specifically, the presence of inflammatory macrophages at acinar cells is tightly linked to intracellular ROS generation and is prerequisite to ADM. Dependent of their resistance to ROS, ADM cells can further progress to PanIN lesions, in which they may proliferate or show a senescent phenotype, or to DCLK1+ cells that can withstand high levels of intracellular ROS. At PanIN lesions and their surrounding microenvironment macrophages are mainly anti-inflammatory and drive lesion growth and fibrosis (10, 18, 24). But they also produce factors that induce ROS in neighboring acinar cells. Therefore, it is fair to say that macrophage-caused oxidative stress is a key driver of events that occur during initiation of pancreatic cancer. However, while a role for ROS in initiation of lesion formation is well established, the roles of ROS in further lesion progression to a more aggressive phenotype is less defined, and it is not fully clear if, and how they contribute to further development of PDA.

## Author contributions

PS: Visualization, Writing – review & editing. HD: Writing – original draft, Writing – review & editing.
